# Rheological decoupling at the Moho and implication to Venusian tectonics

**DOI:** 10.1038/srep04403

**Published:** 2014-03-18

**Authors:** Shintaro Azuma, Ikuo Katayama, Tomoeki Nakakuki

**Affiliations:** 1 Department of Earth and Planetary Systems Science, Hiroshima University, Higashi-Hiroshima 739-8526, Japan

## Abstract

Plate tectonics is largely responsible for material and heat circulation in Earth, but for unknown reasons it does not exist on Venus. The strength of planetary materials is a key control on plate tectonics because physical properties, such as temperature, pressure, stress, and chemical composition, result in strong rheological layering and convection in planetary interiors. Our deformation experiments show that crustal plagioclase is much weaker than mantle olivine at conditions corresponding to the Moho in Venus. Consequently, this strength contrast may produce a mechanical decoupling between the Venusian crust and interior mantle convection. One-dimensional numerical modeling using our experimental data confirms that this large strength contrast at the Moho impedes the surface motion of the Venusian crust and, as such, is an important factor in explaining the absence of plate tectonics on Venus.

Venus is regarded as a twin planet to Earth because of the similar density, size, internal structure, and distance from the Sun of the two planets. However, the Magellan mission to Venus reported no evidence for a global pattern of plate tectonic margins and subduction on Venus, and that the Venusian surface has a nearly uniform age^1^. These observations imply that plate tectonics does not exist on Venus^2^,^3^, although it is the primary control on material and heat circulation on Earth. Consequently, the absence of plate tectonics on Venus as compared with Earth might explain the different evolutionary histories of these otherwise similar rocky planets.

A number of hypotheses have been proposed to explain the absence of plate tectonics on Venus, based on observational data and numerical modeling. Nimmo and McKenzie^4^ suggested that the lithosphere of Venus is unable to create faulting on the scale necessary to form plate boundaries due to the absence of water. In contrast, the large viscosity contrast between a planetary surface and mantle interior is a key factor for stagnant-lid convection, as when the planetary surface is stiff and sluggish as compared with the planetary interior, mantle convection can only occur beneath the lithosphere^5^. In these modeling studies, rheological structure has been shown to play an important role in the evolution of Venus. However, the rheological behavior of planetary interiors is sensitive to temperature, water, and chemical composition, which may all produce strong rheological layering^6^,^7^,^8^,^9^. Rheological decoupling may exist across the chemically distinct layers at the Moho where olivine-rich mantle is overlain by plagioclase-rich crust (Fig. 1). In this study, we carried out laboratory deformation experiments to constrain the nature of rheological layering across the Moho, and we discuss how this may explain the absence of plate tectonics on Venus.

Rheological structure can be inferred from flow laws that represent the strength of solids, which are dependent on strain rate, temperature, and chemical composition^10^,^11^. Mackwell *et al.*^8^ determined the flow law for diabase using rock deformation experiments. These results, when applied to rheological structures in Venus, suggest that a large strength contrast exists between its crust and mantle. In their model, rheological structure was modeled using the power-law creep formula: 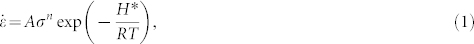
where 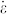 is the strain rate, *A* is a constant, *σ*· is the stress, *n* is the stress exponent, H* is the activation enthalpy, *R* is the gas constant, and *T* is the temperature^12^. This type of flow law is commonly applied to high-temperature creep, although the Peierls mechanism becomes dominant at low temperatures and high stresses^13^. In the Peierls mechanism, the strain rate is exponentially proportional to applied stress as follows: 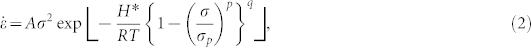
where *σ_p_* is the Peierls stress, and *p* and *q* are non-dimensional parameters that depend on the geometry of kinks^14^. Assuming a thermal gradient of 5–30 K/km and a Moho depth of 7–20 km^15^, the Peierls mechanism is likely to be the dominant mechanism of deformation at the Moho in Venus. Therefore, the power-law creep formula determined by previous studies should not be applied to the rheological structure of the shallow part of Venus. In this study, we conducted two-phase deformation experiments to directly constrain the strength contrast between the crust and mantle at conditions corresponding to the Venusian Moho.

## Results

Deformation experiments were carried out at a pressure of 2.0 GPa and temperatures of 600–1000°C using a solid-medium deformation apparatus at Hiroshima University, Japan. The starting materials of annealed synthetic plagioclase (An_66_Ab_34_) and olivine (Fo_90_Fa_10_) were sandwiched separately between alumina pistons cut at 45° to the maximum compressional direction and oriented to represent the deformation geometry of simple shear (Fig. S2). The experiments were conducted under dry conditions at a constant rate from 6.5 × 10^−6^ to 3.7 × 10^−4^ (s^−1^). Shear strain was measured from the rotation of a nickel strain marker that was initially orientated perpendicular to the shear direction (Fig. 2).

Samples recovered after the deformation experiments showed the development of a crystal-preferred orientation in plagioclase and undulatory extinction in olivine, which suggest that deformation of these minerals was largely controlled by inter-crystal deformation, such as dislocation-controlled creep. The shear strain determined from the rotation angle of the strain marker was up to *γ* = 0.8 for olivine and *γ* = 4.7 for plagioclase, and in nearly simple shear geometry (Table S1). The uncertainty on these shear strain measurements is ca. 10%–20% based on the shape of the strain marker, and the compressional strain was typically <20% of the shear strain (Table S1). The stress values should be identical between the two layers in our experimental assembly. Hence, the strain contrast provides an approximation of the strength contrast at a given stress, temperature, and pressure. Figure 3 shows the strength contrast between plagioclase and olivine as a function of temperature. The strength of plagioclase was lower than that of olivine at any experimental temperature (filled square symbols) and strain rate (filled diamond symbol). These results are the opposite of those predicted from previous studies based on the power-law creep of plagioclase (dashed line), indicating that another deformation mechanism operated under our experimental conditions.

A large amount of extrapolation is needed when applying laboratory data for plastic flow to geological processes, and this extrapolation must be made using an appropriate flow law for the deformation mechanism. Therefore, we determined the dominant deformation mechanism of olivine during our experiments with reference to a deformation mechanism map^11^,^16^,^17^,^18^,^19^,^20^,^21^, which shows that the Peierls mechanism best explains our experimental results at temperatures below 1000°C (Fig. S5). Although the deformation mechanism of plagioclase is not well constrained, our experimental results at low temperatures clearly deviate from the power-law creep determined by previous studies^22^,^23^, in which plagioclase was shown to be stronger than olivine (Fig. 3). This indicates that the Peierls mechanism might also control plagioclase deformation under our experimental conditions. Tsenn and Carter^13^ reported that the Peierls mechanism becomes dominant at low temperatures in materials with relatively strong chemical bonding such as silicates. As such, the strength contrast observed in our experiments is most likely caused by differences in the Peierls stress of olivine and plagioclase.

## Discussion

**Figure 1:**
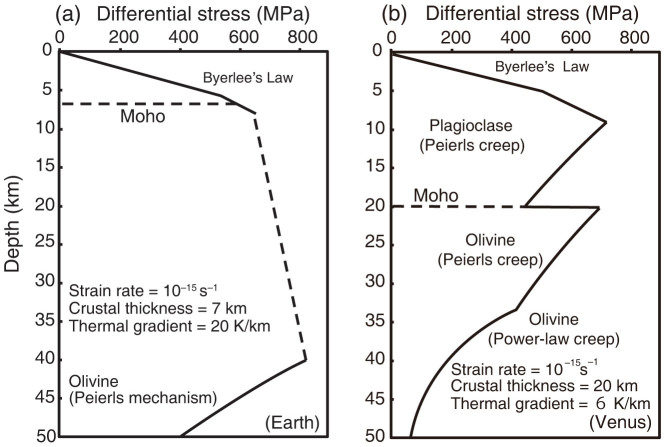
Strength profile models of Earth and Venus. (a) Strength profile of oceanic lithosphere on Earth. The flow law for the Peierls mechanism of olivine in the upper mantle^20^ and a relatively broad brittle-to-plastic transition (dashed line) were adopted from Kohlstedt *et al*^24^. (b) Strength profile model for Venus predicted from our experimental results showing the large strength contrast between the lower crust and upper mantle. These models simulate deformation strength of the Venusian lithosphere at an age of 200 Ma, which is the estimated age of the surface around Artemis Chasma^25^. In this model, the Moho depth was assumed to be the 20 km^15^. Flow law for the Peierls mechanism^20^ and power-law creep^11^,^18^ were used for the mantle. Peierls mechanism for plagioclase, as determined in this study, was applied to the lower crust. The parameters of the flow laws used in this study are summarized in Table S3.

A strength profile was calculated by extrapolating frictional sliding and the viscous flow law of each material to high pressures and temperatures corresponding to a range of depths^9^. Figure 1 shows the strength profiles of Earth and Venus, and highlights that Earth's oceanic lithosphere has no strength contrast between crust and mantle because deformation at the Moho is still in the brittle regime (Fig. 1a)^24^. In this case, the crust and mantle are strongly and dynamically coupled and will move coherently together. In contrast, our experiments show a large strength contrast between the crust and mantle on Venus (Fig. 1b), suggesting that the lower crust may act as a lubricant and therefore inhibit horizontal motion of the entire crust. In this case, the soft lower crustal material may be separated from the convecting mantle and never be able to be subducted into the stiffer mantle material. In support of this hypothesis, we note that Brown and Grimm^25^ suggested that a landform called Artemis Chasma on Venus was produced by underthrusting and strike-slip deformation of the lithosphere, whereas the overlying crustal materials were not subducted. The strength profile from our study indicates that this landform may have resulted from decoupling in the lower crust, which in effect forms a “crustal asthenosphere”. On Earth, similar landforms are observed in continental collision zones, caused by complex deformation of the upper crust, including fold and thrust belts, and strike-slip faulting. The tectonic and rheological models in such zones emphasise the importance of decoupling between the crust and mantle, indicating that the overlying crust was not subducted^26^. Shemenda^27^ carried out analogue subduction experiments incorporating “crustal asthenosphere” for simulating continental collisions on Earth, and produced successive fold and thrust belts in the upper crust, in which the crust could not intrude into the mantle. We consider that a similar phenomenon could have occurred beneath Artemis Chasma in the absence of plate tectonics on Venus. The elastic thickness in Venus has been constrained to a range of 0–20 km in nearly half areas^28^, although Artemis Chasma shows relatively thick elastic layer of 37 km^29^. These estimates of elastic thickness may be caused by the different depth of Moho where decoupling occurs and the overlain crust is nearly stagnant. Mechanical decoupling could cause the decrease of the elastic thickness^30^,^31^.

To evaluate the influence of the strength contrast at the Moho on Venusian tectonics, we numerically analysed viscosity and moving velocity in the lithosphere using a one-dimensional model that simulates an embryonic subduction zone such as Artemis Chasma^25^ (Supplementary Information; Fig. S3). In this analysis, simple shear deformation of the lithosphere beneath the thrust is calculated with fault friction at the lithospheric surface and the motion of the mantle lithosphere. The strength profiles shown in Figure 1 were used for the rheology models of the Venusian lithosphere. The Moho depth in our model was assumed to be 20 km based on gravity and topography data^15^,^28^ (Fig. 1b), although the numerical calculations were also conducted at various crustal thicknesses (20, 45, and 70 km) and produced similar results in each case. Various models with the crust–mantle viscosity contrast ranging from 1 to 10^4^ were tested. Under these conditions, the resultant viscosity and moving velocity depth profiles are shown in Figure 4. The velocity profile demonstrates that crustal motion becomes more with increasing viscosity contrast at the Moho (Fig. 4). Our modeling suggests that a large viscosity difference between the crust and mantle results in a more or less negligible motion of the crustal surface, even though the plate faulting crosses the entire brittle deformation range and the underlying mantle moves at a velocity of 10 cm/yr (Fig. 4). The crustal thickness in Venus is not well constrained; however, our models with different crustal thicknesses results in a similar moving velocity profile in each case (Fig. S4). Although the experimentally determined strain contrast at the Moho is relatively small (~one order of magnitude), once decoupling between the crust and mantle initiates, the motion of the upper crust becomes sluggish and the stress in the crust decrease; consequently, a positive feedback could accelerate the decoupling between the crust and mantle. This results in a convection style with a stagnant crust that is apparently similar to stagnant-lid type convection^32^. Although Aitta^33^ suggested that the small Ra number of Venus played a key role in shaping the different tectonics of Earth and Venus, a viscosity contrast at the Moho facilitated the rheological decoupling that contributed to the absence of plate tectonics on Venus.

In the past, Venus may have had a higher potential temperature than at present. If the potential temperature was 150–200°C higher than at present, which is assumed to be similar to that on Earth during the Archean^34^, the crustal portion would have been weaker than the mantle (Fig. 3). Therefore, decoupling could have occurred at the Moho during Venus' past, suggesting stagnant-lid type convection. However, if the crustal thickness was substantially thinner in the past (<~7 km), deformation in the lower crust would have been controlled by frictional sliding, meaning that a strength contrast at the Moho would have been unlikely to exist. In the later case, the crust might have been coupled with the mantle convection and subducted along with the mantle lithosphere.

We conclude that rheological structure and, in particular, the strength contrast at the Moho, might be a critical factor that has shaped the different tectonic evolutions of Earth and Venus. Note that an application of our laboratory results to the Venusian tectonics requires a large extrapolation; consequently, an understanding of the active deformation mechanism is key to understanding the tectonic evolution of terrestrial planets.

## Methods

**Figure 2:**
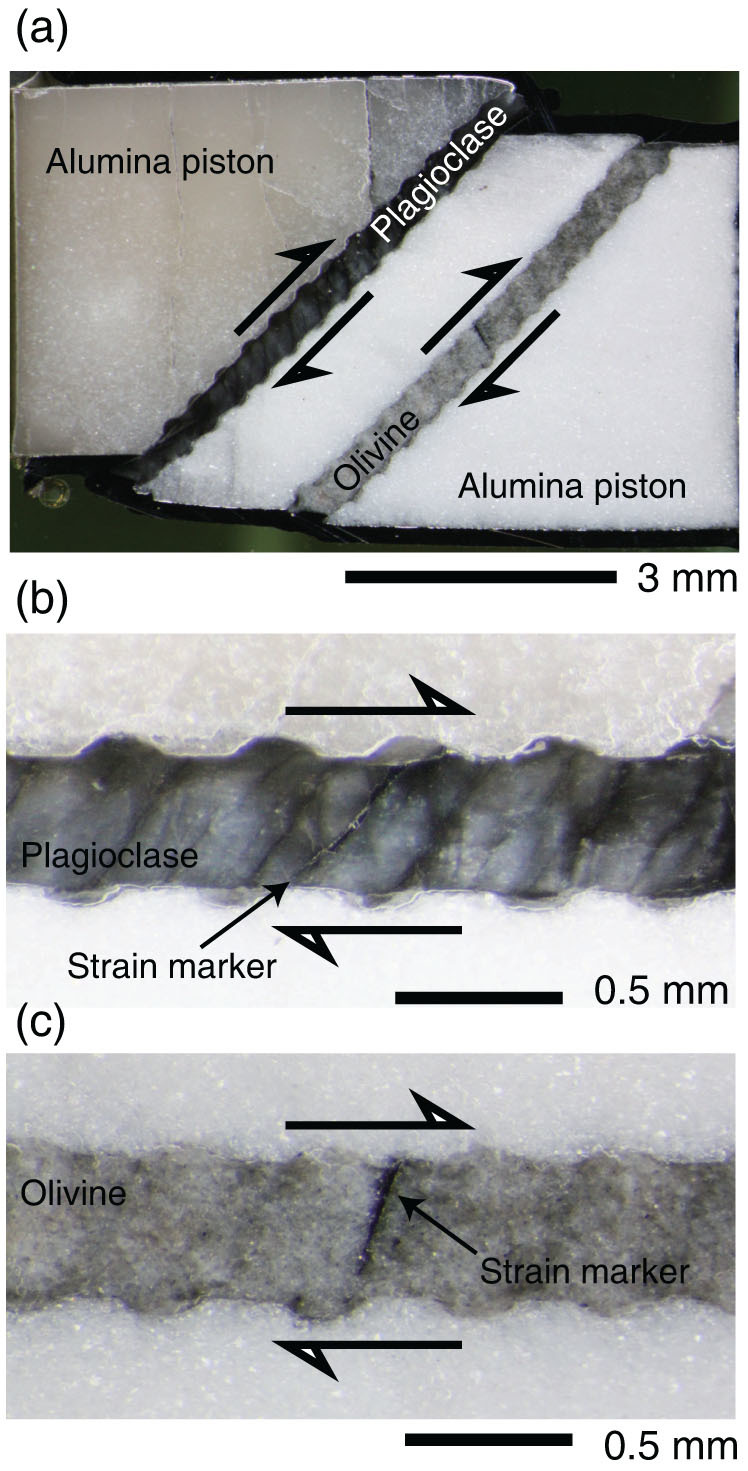
Experimental samples after shear deformation. (a) Olivine and plagioclase samples were sandwiched between alumina pistons, which were cut at an angle of 45° to the maximum compressional direction. The recovered sample was cut along the direction of compression. (b and c) The shear strain of the recovered samples was measured from the rotation of the strain marker that was initially perpendicular to the shear direction. Arrows indicate the sense of shear.

**Figure 3:**
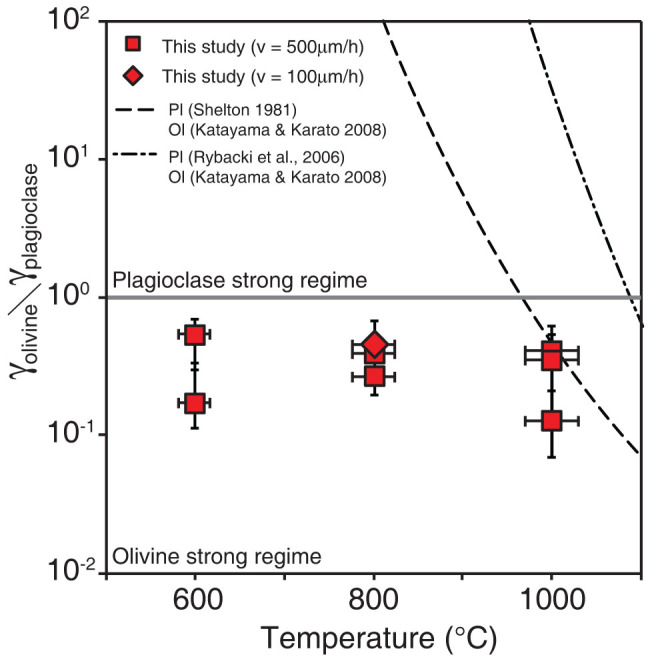
Strain contrast between plagioclase and olivine as a function of temperature. Square and diamond symbols denote the experimental results for strain rates of ~10^−5^ and ~10^−6^ s^−1^, respectively. Dashed lines represent the Strain contrast calculated from Peierls mechanism for olivine^20^ and power-law creep of plagioclase^23^,^35^ at a strain rate of 10^−5^ s^−1^. The horizontal error–bars come from temperature gradient within samples.

**Figure 4:**
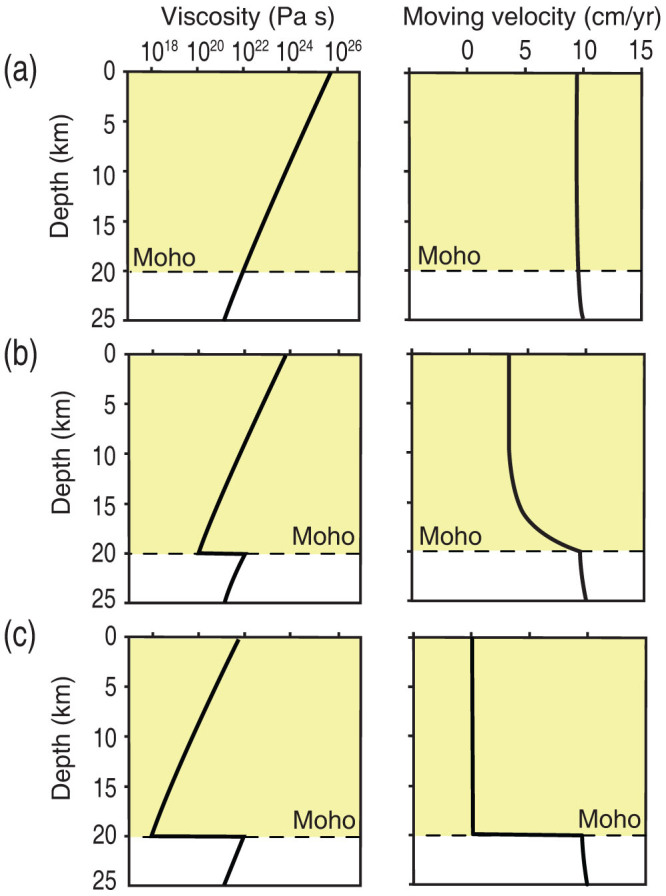
Viscosity (left) and moving velocity (right) profiles calculated using a one-dimensional deformation model for the Venusian lithosphere. The numerical modeling results based on our experimental data show that the planetary surface becomes immobile when there is a large strength contrast at the Moho. (a) Model with no strength contrast at the Moho. (b and c) Model with a viscosity contrast of 10^2^ and 10^4^ Pa s at the Moho, respectively. The internal velocities obtained for each model are shown assuming that the crustal thickness is 20 km and the bottom (mantle) velocity is 10 cm/yr. In case c, the velocity is truncated at zero as the negative value of the crustal moving velocity means that the crust becomes immobile with a smaller fault friction than that used in this study.

Deformation experiments were carried out at a pressure of 2.0 GPa and temperatures of 600–1000°C using a solid-medium deformation apparatus at Hiroshima University, Japan. The starting materials of annealed synthetic plagioclase (An_66_Ab_34_) and olivine (Fo_90_Fa_10_) were sandwiched separately between alumina pistons cut at 45° to the maximum compressional direction and oriented to represent the deformation geometry of simple shear (Fig. S2). The experiments were conducted under dry conditions, and the water contents of the starting materials were verified by Fourier-transform infrared spectroscopy as being ca. <30 ppm H/Si for both minerals (Fig. S1). Tiny grooves were made at the interface between the sample and piston to prevent slip during deformation. A nickel jacket surrounded these materials, and the oxygen fugacity was buffered by the Ni–NiO reaction. The pressure was first raised to 0.1 GPa, and then the temperature was increased at a rate of ~15°C/min to 400°C. The pressure was then increased to 2.0 GPa and the temperature was increased to the desired value. Temperature was monitored by two Pt/Rh thermocouples placed close to the sample. When pressure and temperature had attained the desired values, a piston was advanced at a constant rate from 6.5 × 10^−6^ to 3.7 × 10^−4^ (s^−1^). The samples were rapidly quenched by switching off the thermocontroller when the deformation experiments were finished, and the pressure was then reduced. Shear strain was measured from the rotation of a nickel strain marker that was initially orientated perpendicular to the shear direction (Fig. 2).

## Author Contributions

S.A. and I.K. planned the project and performed the experiments. T.N. advised and helped with the programming. All authors discussed the results and interpretation of the data.

## Supplementary Material

Supplementary Information Supplementary information

## References

[b1] Turcotte D. L., Morein G., Roberts D. & Malamud B. D. Catastrophic resurfacing and episodic subduction on Venus. Icarus 139, 49–54 (1999).

[b2] Kaula W. M. & Phillips R. J. Quantitative tests for plate tectonics on Venus. Geophys. Res. Lett. 8, 1187–1190 (1981).

[b3] Phillips R. J. & Malin M. C. Tectonics of Venus. Ann. Rev. Earth Planet. Sci. 12, 411–443 (1984).

[b4] Nimmo F. & McKenzie D. Volcanism and tectonics on Venus. Ann. Rev. Earth Planet. Sci. 26, 23–51 (1998).

[b5] Solomatov V. S. & Moresi L. N. Stagnant lid convection on Venus. J. Geophys. Res. 101, 4737–4753 (1996).

[b6] Zuber M. T. Constraints on the lithospheric structure of Venus from mechanical models and tectonic surface features. J. Geophys. Res. 92, E541–E551 (1987).

[b7] Kaula W. M. Venus reconsidered. Science 270, 1460–1464 (1995). 7491490 10.1126/science.270.5241.1460

[b8] Mackwell S. J., Zimmerman M. E. & Kohlstedt D. L. High-temperature deformation of dry diabase with application to tectonics on Venus. J. Geophys. Res. 103, 975–984 (1998).

[b9] Burgmann R. & Dresen G. Rheology of the lower crust and upper mantle: Evidence from rock mechanics, geodesy, and field observations. Ann. Rev. Earth Planet. Sci. 36, 531–567 (2008).

[b10] Frost H. J. & Ashby M. F. Deformation-Mechanism Maps: The Plasticity and Creep of Metals and Ceramics. (Pergamon Press, Oxford, 1982).

[b11] Karato S. & Jung H. Effects of pressure on high-temperature dislocation creep in olivine. Philos. Mag. A83, 401–414 (2003).

[b12] Poirier J. P. Creep of Crystals. (Cambridge University Press, New York, 1985).

[b13] Tsenn M. C. & Carter N. L. Upper limits of power law creep of rocks. Tectonophys. 136, 1–26 (1987).

[b14] Kocks U. F., Argon A. S. & Ashby M. F. Thermodynamics and kinetics of slip. Prog. Mater. Sci. 19, 1–291 (1975).

[b15] James P. B., Zuber M. T. & Phillips R. J. Crustal thickness and support of topography on Venus. J. Geophys. Res. 118, 859–875 (2013).

[b16] Evans B. & Goetze C. The temperature variation of hardness of olivine and its implication for polycrystalline yield stress. J. Geophys. Res. 84, 5505–5524 (1979).

[b17] Karato S., Paterson M. S. & Fitzgerald J. D. Rheology of synthetic olivine aggregates: Influence of grain size and water. J. Geophys. Res. 91, 8151–8176 (1986).

[b18] Mei S. & Kohlstedt D. L. Influence of water on plastic deformation of olivine aggregates 2. Dislocation creep regime. J. Geophys. Res. 105, 21471–21481 (2000).

[b19] Hirth G. & Kohlstedt D. Rheology of the upper mantle and the mantle wedge: A view from the experiments. Geophys. Monogr. 138, 83–105 (2003).

[b20] Katayama I. & Karato S. Low-temperature, high-stress deformation of olivine under water-saturated conditions. Phys. Earth Planet. Inter. 168, 125–133 (2008).

[b21] Demouchy S. Tommasi A., Ballaran T. B. & Cordier P. Low strength of Earth's uppermost mantle inferred from tri-axial deformation experiments on dry olivine crystals. Phys. Earth Planet. Inter. 220, 37–49 (2013).

[b22] Rybacki E. & Dresen G. Dislocation and diffusion creep of synthetic anorthite aggregates. J. Geophys. Res. 105, 26017–26036 (2000).

[b23] Rybacki E., Gottschalk M., Wirth R. & Dresen G. Influence of water fugacity and activation volume on the flow properties of fine-grained anorthite aggregates. J. Geophys. Res. 111, B03203, 10.1029/2005JB003663 (2006).

[b24] Kohlstedt D. L., Evans B. & Mackwell S. J. Strength of lithosphere: Constraints imposed by laboratory experiments. J. Geophys. Res. 100, 17587–17602 (1995).

[b25] Brown C. D. & Grimm R. E. Recent tectonic and lithospheric thermal evolution of Venus. Icarus 139, 40–48 (1999).

[b26] Willett S., Beaumont C. & Fullsack P. Mechanical model for the tectonics of doubly vergent compressional orogens. Geology 21, 371–374 (1993).

[b27] Shemenda A. I. Subduction: Insights from Physical Modelling. (Dordrecht, Kluwer Academic Publishers, 1994).

[b28] Anderson F. S. & Smrekar S. E. Global mapping of crustal and lithospheric thickness on Venus. J. Geophys. Res. 111, E08006, 10.1029/2004JE002395 (2006).

[b29] Sandwell D. T. & Schubert G. Flexural ridges, trenches, and outer rises around coronae on Venus. J. Geophys. Res. 97, 16069–16083 (1992).

[b30] Burov E. B. & Diament M. The effective elastic thickness (*T_e_*) of continental lithosphere: What does it really mean? J. Geophys. Res. 100, 3905–3927 (1995).

[b31] Brown C. D. & Phillips R. J. Crust–mantle decoupling by flexure of continental lithosphere. J. Geophys. Res. 105, 13221–13237 (2000).

[b32] Moresi L. N. & Solomatov V. S. Mantle convection with a brittle lithosphere: thoughts on the global tectonic styles of the Earth and Venus. Geophys. J. Int. 133, 669–682 (1998).

[b33] Aitta A. Venus' internal structure, temperature and core composition. Icarus 218, 967–974 (2012).

[b34] Komiya T. Material circulation model including chemical differentiation within the mantle and secular variation of temperature and composition of the mantle. Phys. Earth Planet. Inter. 146, 333–367 (2004).

[b35] Shelton G. L. Experimental deformation of single phase and polyphase crustal rocks at high pressures and temperatures., Ph.D. thesis, Brown Univ., Providence, R.I. (1981).

