# Supplementary material for: Rheological decoupling at the Moho and implication to Venusian tectonics
Source: Sci Rep. 2014 Mar 18;4:4403. doi: 10.1038/srep04403 (PMC3957145; doi:10.1038/srep04403)
Supplement: Supplementary Information [file srep04403-s1.pdf]

Supplementary information

Rheological decoupling at the Moho and implication to Venusian tectonics

Shintaro Azuma[1]*, Ikuo Katayama[1], and Tomoeki Nakakuki[1]

[1] *Department of Earth and Planetary Systems Science, Hiroshima University, Higashi-Hiroshima 739-8526, Japan*

Experimental methods

Dry hot-pressed starting materials of olivine and plagioclase were prepared. Firstly, inclusion-free crystals of plagioclase ($An_{66}Ab_{34}$) and olivine ($Fo_{90}Fa_{10}$) were carefully selected and crushed using a vibrational mill. Powders of plagioclase and olivine were then cold-pressed separately at P = ~60 MPa without water. These materials were annealed at P = 196 MPa and T = 1100–1250°C for ~ 6 h using a gas-medium apparatus at the Institute for Study of the Earth's Interior, Okayama University, Japan. The hot-pressed samples showed nearly random orientations of crystals and had an average grain size of ~15 μm and ~20 μm for plagioclase and olivine, respectively. Each sample was dried in an oven at 500°C for 48 h. We verified the water contents of the

hot-pressed samples using Fourier-transform infrared (FTIR) spectroscopy as being ca. <30 ppm H/Si for both materials. It is difficult to completely remove water from these materials. However, Fourier-transform infrared (FTIR) spectroscopy detected no significant hydroxyl-related peaks (Fig. S1). In addition, water contents of 30 ppm H/Si have only minor rheological effects, as reported by Karato and Jung[11]. Simple shear deformation experiments were performed using a modified Griggs-type solid-medium deformation apparatus at P = 2.0 GPa, T = 600–1000°C, and at a constant strain-rate from 6.5×10^{-6} to 3.7×10^{-4} (s^{-1}). Thin slices of plagioclase and olivine samples (~0.45 mm thick) were separately sandwiched between alumina pistons in a simple shear geometry (Fig. S2). The pressure was first raised to 0.1 GPa, and then the temperature was increased at a rate of ~15°C/min to 400°C. The pressure was then increased to 2.0 GPa and the temperature was increased to the desired value. Temperature was monitored by two Pt/Rh thermocouples placed close to the sample. After the temperature stabilized, the piston was advanced. The samples were rapidly quenched by switching off the thermocontroller when the deformation experiments were finished, and the pressure was then reduced. The samples were sealed by Ni capsule to decrease

the thermal gradient. The thermal gradient between samples was evaluated by changing the positions of thermocouples. The temperature is proportional to the distance from the central position of heater in our experimental configuration ($Tg = 0.03 \times Tc$, Tg ; Thermal gradient Tc ; central temperature of graphite heater) (Fig. 3). The strain contrast was measured from the rotation of the nickel strain marker in the recovered samples (Fig. 2). We determined the strength contrast from the central portion of the strain-marker without considering the deformation around the interface of the samples and alumina piston in order to exclude the wall effect of the piston. The strain rate was calculated from the obtained strain and the duration of the deformation. The experimental conditions and results are summarised in the Supplementary Table 1.

Numerical modeling

Our numerical modeling considers the viscosity and moving velocity depth profiles of the lithosphere under a thrust fault, which simulates an embryonic subduction zone. One-dimensional simple shear deformation in the lithosphere was calculated under the boundary condition with thrust shear traction and the moving velocity of the base of the

lithosphere. The Moho depth was assumed to be 20 or 7 km (Fig. 1b). We considered the thrust to occur between colliding lithospheres (Fig. S3). We assumed that friction at the thrust section generates shear traction that resists the lithospheric subduction. In the deeper part of the section, shear stress was estimated to be much smaller due to viscous deformation in the crust and mantle at high temperature. In our modeling, the shear traction value (τ) was set to be equal to the maximum friction in the deepest part of the thrust, which is expressed by a Byerlee's law as

$$\tau = f_c \sigma_n = f_c \rho g l_d$$

where f_c is the frictional coefficient, σ_n is the normal stress, ρ is the density, g is the gravitational acceleration, and l_d is the maximum depth of the thrust. The value of f_c is assumed to be 0.85 and the value of l_d depends on the crustal thickness of the model (Fig. 1). In the model with a depth of 7 km to the Moho, the value of l_d was set to 13 km, whereas l_d was assumed to be 6 km in the case of a Moho depth of 20 km (Fig. 1b). The velocity in the mantle lithosphere at depths of 10 and 25 km for Moho depths of 7 and 20 km, respectively, was assumed to be 10 cm/yr.

We calculated four cases with variable viscosity differences between the crust and mantle in the lithosphere. The first model has no viscosity contrast at the Moho (Fig. 4a and S4a). The second and third models have differences in viscosity of 10^2 and 10^4 Pa s at the Moho (Fig. 4b, 4c, S4b, and S4c). Under these stress and velocity boundary conditions, the entire lithosphere has a constant stress value. Therefore, the viscosity (η) of the crust and mantle is given by an Arrhenius-type equation as follows:

$$\eta = A' \exp\left(\frac{E^* + PV^*}{RT}\right)$$

where A′ is a pre-exponential factor, E^* is the activation energy, P is the hydrostatic pressure, V^* is the activation volume, R is the gas constant, and T is temperature. The viscosity contrast between the crust and mantle is introduced by changing A′ of the crust. The activation energies of the crust and mantle are assumed to be 460 and 495 kJ/mol, respectively, which are smaller than those for power-law creep formula that mimics the Peierls mechanism at constant stress.

The temperature gradient of the lithosphere was set to be constant at 6 K/km. This value was calculated from the shallow temperature of a lithosphere with an age of 200 Ma,

which was estimated for Artemis Chasma[25] using a half-space cooling model[36]. The thermal gradient used in our study is comparable to the thermal gradient of Artemis Chasma reported in previous studies[29,37]. Our calculation results for the velocity in the lithosphere are largely insensitive to the temperature gradient.

The moving velocity at each depth was calculated from the one-dimensional Navier–Stokes equation as follows:

$$\frac{d}{dz}\left[\eta\left(\frac{du}{dz}\right)\right]=0$$

where u is the moving velocity. The z-direction was chosen to be the direction towards the lithospheric interior (Fig. S3). The equation was solved by the finite volume method. The parameters for the numerical models are summarised in Supplementary Table 2.

Although the numerical calculations were conducted at various crustal thicknesses (20, 45, and 70 km), our conclusions are independent of the assumed crustal thickness (i.e., the strength contrast at the Moho efficiently decreases the plate velocity).

Reference

36. Parsons, B. & Sclater J. G. An analysis of the variation of ocean floor bathymetry and heat flow with age. *J. Geophys. Res.* **82**, 803–827 (1977).

37. Brown, C. D. & Grimm, R. E. Lithospheric rheology and flexure at Artemis Chasma, Venus. *J. Geophys. Res.* **101**, 12697–12708 (1996).

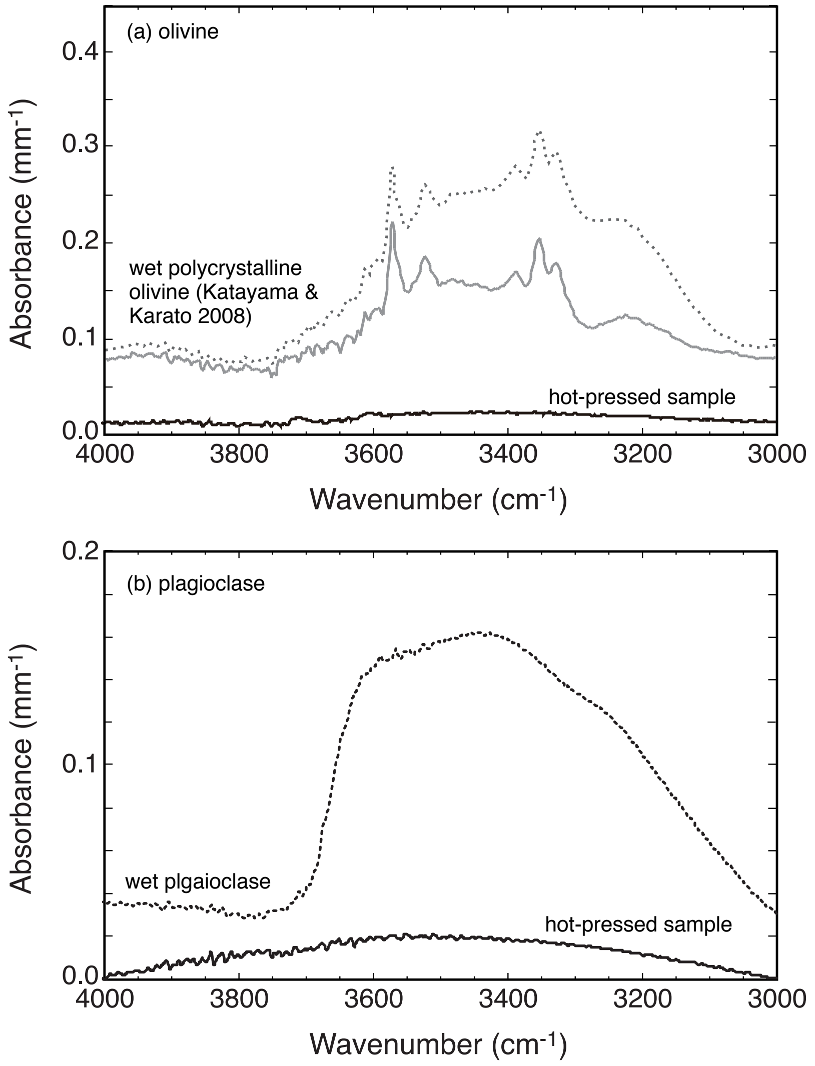

Figure S1. Infrared spectra of olivine and plagioclase. (a) Olivine shows no sharp hydroxyl peaks at 3360, 3530, or 3570 cm^{-1} that would be related to the structurally incorporated hydroxyl. The IR spectra of the hot-pressed starting material and the wet polycrystalline crystal[20] are shown for comparison. (b) Plagioclase also has no sharp hydroxyl peaks that might be related to the presence of hydroxyl.

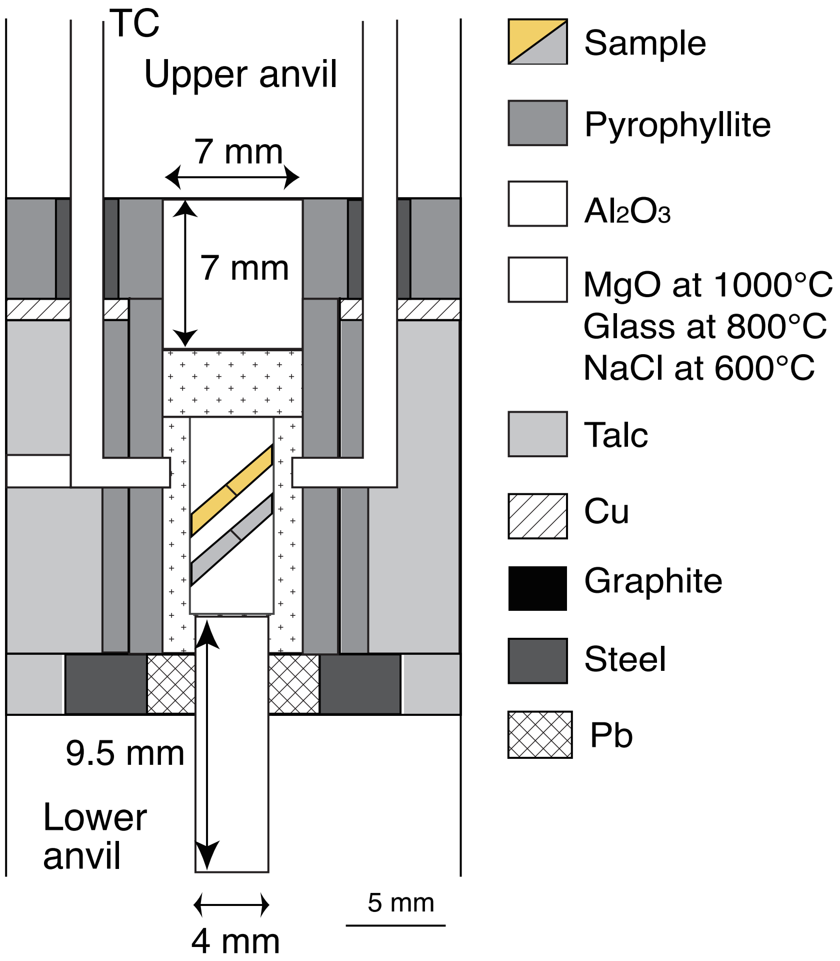

Figure S2. The experimental assembly of the shear deformation experiments using a solid-medium apparatus. Two-phase deformation experiments were conducted in this study. The plagioclase and olivine samples were placed into the alumina piston, which was cut at 45° to the maximum compressional direction.

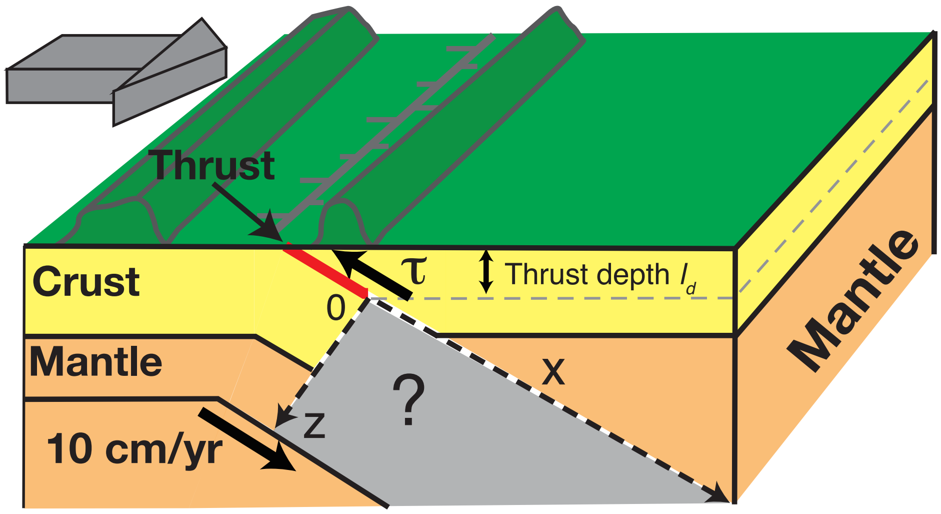

Figure S3. Schematic illustration of our numerical model. The x-axis is in the direction of the thrust, and the z-axis is perpendicular to the thrust. The crustal thickness was set to 7 or 20 km. We calculated simple shear deformation between the plate surface and plate bottom. The surface boundary receives shear traction due to fault friction. The bottom boundary was set to the mantle lithosphere interior at z = 10 and 25 km for crustal thicknesses of 7 and 20 km, respectively, and moves with a velocity of 10 cm/yr.

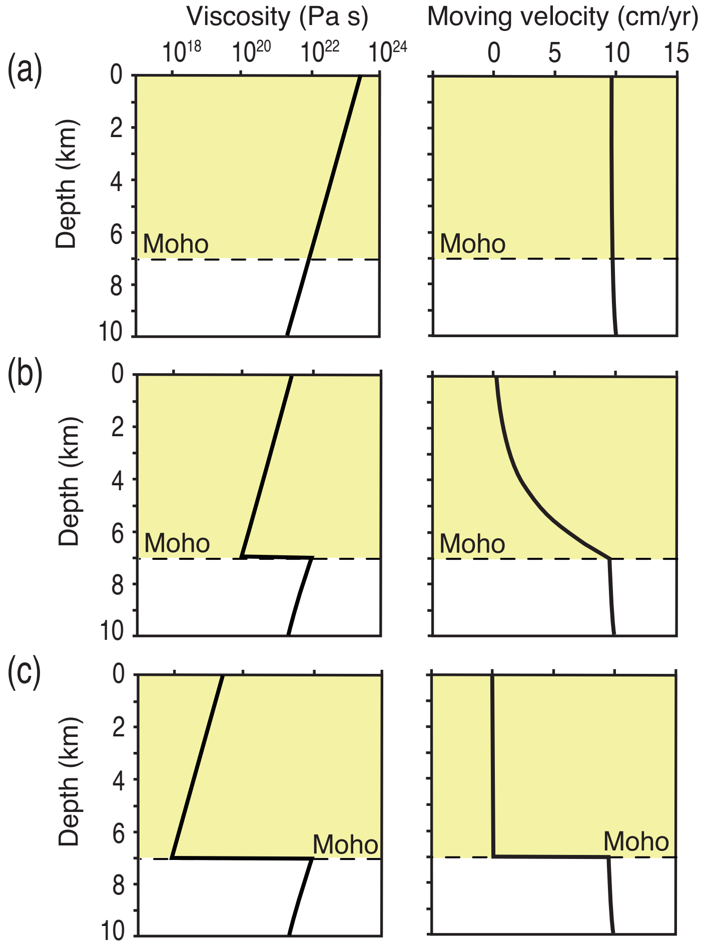

Figure S4. Viscosity (left) and moving velocity (right) profiles calculated using the one-dimensional deformation model for the Venusian lithosphere at a crustal thickness of 7 km. All parameters apart from the crustal thickness are the same as those for the models shown in Figure 4. The truncation of the negative velocity at zero in case c in this figure is the same as for case c in Figure 4.

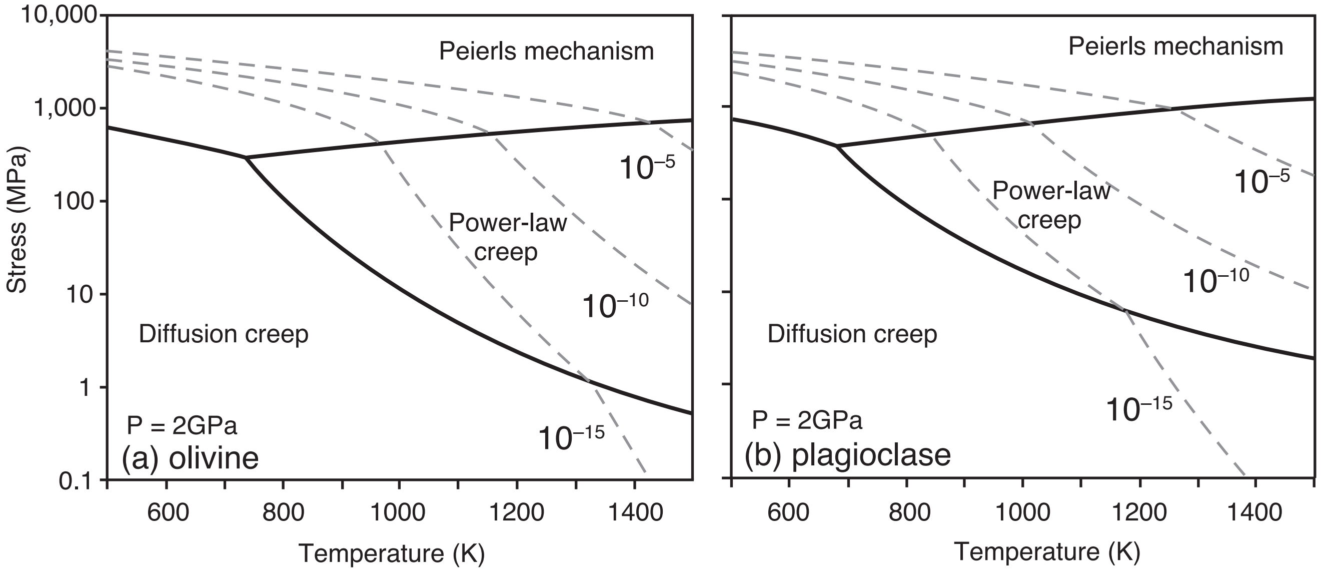

Figure S5. Deformation mechanism map for dry olivine and dry plagioclase. The stress is plotted as a functions of temperature at P = 2.0 GPa and grain size of 1.0 mm. The thick solid line represents the transition of the deformation mechanism and the dashed lines show different strain-rates at 10^{-5}, 10^{-10}, and 10^{-15}. (a) The transitions between the deformation mechanism of olivine were calculated for diffusion creep[19], power-law creep[11,18], and Peierls mechanism[20]. (b) The transitions between the deformation mechanisms of plagioclase were calculated for diffusion creep[22], power-law creep[35], and Peierls mechanism (this study).

Table S1. Experimental conditions and results

Sample no.	Condition				Results				
	Starting material	P (GPa)	T (°C)	Speed (μm/h)	grain size (μm)	compression strain	γ obtained	Strain-rate (/s)	η_{pl}/η_{ol}
Hi-446	Dry olivine	2.0	600	500	16	0.04	0.2 ± 0.1	2.0E-05	0.24
	Dry plagioclase				14	0.10	1.0 ± 0.1	8.5E-05	
Hi-454	Dry olivine	2.0	600	500	16	0.01	0.4 ± 0.1	3.1E-05	0.55
	Dry plagioclase				15	0.20	0.7 ± 0.2	5.6E-05	
Hi-443	Dry olivine	2.0	800	500	19	0.22	0.3 ± 0.1	2.3E-05	0.27
	Dry plagioclase				14	0.09	1.1 ± 0.1	8.7E-05	
Hi-435	Dry olivine	2.0	800	500	19	0.13	0.3 ± 0.1	2.2E-05	0.40
	Dry plagioclase				16	0.22	0.6 ± 0.1	5.6E-05	
Hi-452	Dry olivine	2.0	1000	500	18	0.10	0.6 ± 0.2	4.7E-05	0.13
	Dry plagioclase				16	0.04	4.7 ± 0.9	3.7E-04	
Hi-456	Dry olivine	2.0	1000	500	17	0.25	0.8 ± 0.1	7.9E-05	0.41
	Dry plagioclase				15	0.15	2.0 ± 0.2	1.9E-04	
Hi-458	Dry olivine	2.0	1000	500	nd	0.24	0.8 ± 0.2	8.8E-05	0.36
	Dry plagioclase				nd	0.08	2.4 ± 0.4	2.5E-04	
Hi-459	Dry olivine	2.0	800	100	nd	0.14	0.5 ± 0.1	6.5E-06	0.46
	Dry plagioclase				nd	0.08	1.1 ± 0.2	1.4E-05	

Table S2. Parameters in numerical calculation

Symbol		Explanations	Value
Parameter of flow law			
Crust	A'	Pre-exponential factor	
		(Crustal thickness 7 km; Fig. 4A)	1.2×10^{-9} Pa s
		(Crustal thickness 7 km; Fig. 4B)	1.2×10^{-11} Pa s
		(Crustal thickness 7 km; Fig. 4C)	1.2×10^{-13} Pa s
		(Crustal thickness 20 km; Fig. S4A)	2.5×10^{-7} Pa s
		(Crustal thickness 20 km; Fig. S4B)	2.5×10^{-9} Pa s
		(Crustal thickness 20 km; Fig. S4C)	2.5×10^{-11} Pa s
	E^*	Activation energy	460 kJ mol
	V^*	Activation volume	24 $cm^3\ mol^{-1}$
Mantle	A'	Pre-exponential factor	
		(Crustal thickness 7 km)	7.8×10^{-12} Pa s
		(Crustal thickness 20 km)	4.0×10^{-9} Pa s
	E^*	Activation energy	495 $kJ\ mol^{-1}$
	V^*	Activation volume	14 $cm^3\ mol^{-1}$
Boundary condition			
	τ	Shear traction at surface	
		(Crustal thickness 7 km)	284 MPa
		(Crustal thickness 20 km)	153 MPa
	U_b	Velocity at bottom	10 cm $year^{-1}$
Physical property			
	ρ_c	Crustal density	2900 kg / m^3
	ρ_m	Mantle density	3300 kg / m^3
	T_s	Surface temperature	743 K
	T_g	Thermal gradient	6 K km^{-1}
	g	Gravity acceleration	8.87 m s^{-2}
	R	Gas constant	8.3144 J $K^{-1}\ mol^{-1}$
	F_c	Frictional coefficient	0.85

Parameters for the mantle were taken from *Mei and Kohlstedt* (2000)[18] and *Karato and Jung* (2003)[11].

Table S3. Parameters of flow law of olivien and plagioclase used in this study.

	Deformation mechanism	logA (S⁻¹MPa⁻ᵐ)	n (m)	r	σ_p (MPa)	E (kJ/mol)	V (cm³/mol)	Reference
Plagioclase wet	Diffusion creep	-0.7	1 (3)	1	–	159	38	Rybacki et al.(2006)
	Dislocation creep	-5.6	3.9	–	–	235	–	Shelton (1981)
		0.2	3	1		345	38	Rybacki et al.(2006)
	Peierls creep	-1.2	2	–	3410	235	–	This study & Azuma et al., (2010)
Plagioclase dry	Diffusion creep	5.1	1 (3)	–	–	268	–	Rybacki & Dresen (2000)
	Dislocation creep	0.9	4	–	–	431	–	Shelton (1981)
		12.7	3	–	–	641	24	Rybacki & Dresen (2006)
	Peierls creep	3.48	2	–	9831	431	–	This study
Olivine wet	Diffusion creep	6.0	1 (3)	1	–	335	4	Hirth & Kohlstedt (2003)
	Dislocation creep	0.56	3	1.2	–	410	11	Karato & Jung (2003)
	Peierls creep	0.67	2	1.2	2870	410	11	Katayama & Karato (2008)
Olivine dry	Diffusion creep	9.2	1 (3)	–	–	375	5	Hirth & Kohlstedt (2003)
	Dislocation creep	6.1	3	–	–	510	14	Karato & Wu (1993)
	Peierls creep	6.27	2	–	9600	510	14	Evans & Goetze (1993)
